# Searching for Lower Female Genital Tract Soluble and Cellular Biomarkers: Defining Levels and Predictors in a Cohort of Healthy Caucasian Women

**DOI:** 10.1371/journal.pone.0043951

**Published:** 2012-08-31

**Authors:** Jordan K. Kyongo, Vicky Jespers, Odin Goovaerts, Johan Michiels, Joris Menten, Raina N. Fichorova, Tania Crucitti, Guido Vanham, Kevin K. Ariën

**Affiliations:** 1 Virology Unit, Division of Microbiology, Department of Biomedical Sciences, Institute of Tropical Medicine, Antwerp, Belgium; 2 ITM HIV/AIDS Centre, Department of Public Health, Institute of Tropical Medicine, Antwerp, Belgium; 3 Immunology Unit, Division of Microbiology, Department of Biomedical Sciences, Institute of Tropical Medicine, Antwerp, Belgium; 4 Clinical Trials Unit, Department of Clinical Sciences, Institute of Tropical Medicine, Antwerp, Belgium; 5 Laboratory of Genital Tract Biology, Department of Obstetrics, Gynaecology and Reproductive Biology, Brigham and Women's Hospital, Harvard Medical School, Boston, Massachusetts, United States of America; 6 HIV/STI Reference Laboratory, Department of Clinical Sciences, Institute of Tropical Medicine, Institute of Tropical Medicine, Antwerp, Belgium; 7 Faculty of Pharmaceutical, Veterinary and Biomedical Sciences, University of Antwerp, Antwerp, Belgium and Faculty of Medicine and Pharmacology, University of Brussels, Brussels, Belgium; Rush University, United States of America

## Abstract

**Background:**

High concentrations of pro-inflammatory cytokines have been previously observed in the genital fluids of women enrolled in microbicide trials and may explain observed increased HIV transmission in some of these trials. Although the longitudinal nature of these studies allows within-subject comparisons of post-product levels to baseline levels, the fact that the physiologic variations of these cytokines and other markers of immune activation are not fully defined in different populations, makes it difficult to assess changes that can be directly attributed to microbicide use as opposed to other biological and behavioural factors.

**Methods:**

Cervicovaginal lavage samples were collected from 30 healthy Caucasian and assayed for concentrations of ten cytokines/chemokines, total protein content and two antimicrobial proteins using a multiplex immunoassay and ELISA. Cellular markers were characterized by flow cytometry on mononuclear cells collected from the endocervix using flocked swabs. Bacterial quantification was performed using quantitative PCR.

**Results:**

Ectopy, menstrual cycle phase, prostate-specific antigen and presence of leucocytes in endocervical cells' supernatant were associated with the concentrations of cyto-/chemokines in cervicovaginal secretions. Approximately 3% of endocervical cells collected were monocytes of which a median of 52% (SD  = 17) expressed both CD4 and CCR5 markers. Approximately 1% of the total cells were T-cells with a median of 61% (SD  = 10) CD4 and CCR5 expression. Around 5% of the monocytes and 16% of the T-cells expressed the immune activation marker HLA-DR. Higher percentages of T-cells were associated with greater quantities of IL-1RA, GM-CSF and elafin.

**Conclusion:**

We demonstrate the presence of selected soluble and cellular immune activation markers and identify their predictors in the female genital tract of healthy women. Future clinical trials should consider ectopy, sexual activity, menstrual cycle phase and presence of bacterial species as possible confounders when evaluating the possible inflammatory effects of microbicide compounds.

## Introduction

In the HIV prevention campaign, safe and effective anti-HIV microbicides would offer a discrete protection option when applied vaginally by women before and/or after sexual intercourse but also rectally by men who have sex with men (MSM). Though modest, recent success with the CAPRISA 004 tenofovir gel trial that conferred 39% protection against HIV infection in sexually active women in South Africa [1] provided proof of concept and a much needed impetus in the field of anti-HIV microbicide development although a subsequent study in a different population (VOICE) failed to show effectiveness of the vaginal tenofovir gel [2]. Oral antiretrovirals (ARVs) have also been shown to be effective pre-exposure chemoprophylactic agents in MSM [3] as well as in heterosexual discordant couples [4]. Previously, clinical trials with first generation vaginal microbicide compounds including surfactants and entry/fusion inhibitors either showed no effectiveness or resulted in an increased risk of HIV infection in the subjects who used them although they had proven antiviral activity *in vitro* [5], [6], [7]. It was postulated that the increased infection risk could have been partially due to microbicide-induced mucosal inflammation that results in attraction and activation of CD4+ immune cells, which are prime targets for HIV infection. Excessive inflammation and compound toxicity could also compromise the cervical and vaginal epithelial integrity exposing sub-epithelial target cells like dendritic cells (DC's), CD4+ T-lymphocytes and macrophages to HIV [8], [9]. Either way, a better understanding of the mucosal immunity and its modulating factors in different human populations is essential for designing better anti-HIV microbicides and for characterizing drug effects on HIV transmission.

Efforts are therefore underway to define soluble and cellular biomarkers that could be used in microbicide trials to assess sub-clinical mucosal inflammation and hence increase product safety [10], [11], [12]. Quantification of soluble biomarkers including pro- and anti-inflammatory cytokines found in female genital tract (FGT) secretions has been done in cervicovaginal lavage (CVL) samples or endocervical secretions (ECS) samples collected using different types of swabs or sponges. Considerable data has been generated on cytokine concentrations in the FGT secretions of HIV-positive [13], [14], high risk HIV-negative [13], low risk HIV-negative [13], microbicide trial participants [15] and even healthy women [12]. However, methodological variations in sample collection, processing and assay still present a challenge for comparison of data between different studies [16], a challenge also encountered in cytokine measurement in serum and plasma samples [17], [18]. Although attempts have been made to standardize cytokine measurement in the vaginal and blood serum context [19] and baseline confidence intervals have been published for some of them in CVLs [19], normative values for these cytokines and other markers of immune activation have not been defined in all populations making it hard to assess cytokine variations that can be attributed to microbicide use as opposed to age, hormonal changes during the menstrual cycle, vaginal tract infection and exposure to semen among other factors.

Definition of cellular markers of immune activation in the FGT of healthy women is also of paramount importance not only because some of these cells are targets for initial HIV infection, but in addition, their recruitment to mucosal surfaces propagates local HIV replication and subsequent systemic dissemination due to normal immune trafficking mechanisms [20], [21]. The healthy female genital tract is also home to certain bacterial species that create an acidic environment hostile to pathogens. Imbalance in the vaginal microbiome can result in bacterial vaginosis (BV) that has been associated with greater susceptibility to HIV infection [22]. To our knowledge, there is no study to date that correlates the above mentioned soluble markers of immune activation with cellular immune activation markers, the vaginal microbiome and clinical data all of which are part of the complex FGT environment targeted in microbicide trials. Generation of data on the ranges of cytokines/chemokines, local cell populations and the factors that affect their expression in healthy women is needed for assessing the safety of future microbicide candidates.

In this study, we report concentrations of selected cytokines, chemokines and growth factors as well as β-defensin and the anti-protease elafin in CVLs from healthy women representative of a typical early phase I trial population and establish clinical factors associated with their immunoassay detection. We also characterize by flow cytometry the proportion of T-cells and monocytes, as well as their expression of the activation marker HLA-DR and the HIV co-receptor CCR5 in the endocervical canal of these women. The association between the vaginal microbiome composition with these soluble and cellular factors is also examined.

## Methods

### Ethical statement

IRB approval was obtained from the Institute of Tropical Medicine and from the Ethics Committee at the University Hospital of Antwerp. All clinical investigations were conducted according to the principles expressed in the Declaration of Helsinki. All study participants gave their written informed consent.

### Study subjects

Thirty women aged between 19 and 38 years were recruited at the Institute of Tropical Medicine in Antwerp, Belgium using a previously described recruitment strategy for a classical healthy population for a phase I microbicide trial [23]. These women were not pregnant, did not use any hormonal contraception for the duration of the study, they did not have vaginal infections at screening and had a regular menstrual cycle. Sexual activity was allowed and condoms were provided. Women were screened and then scheduled for five follow-up visits on days 7 (+/− 2 in the follicular phase) and 21 (+/− 2 in the luteal phase) of the three subsequent menstrual cycles. At each visit, a written questionnaire was completed by the women about their sexual activity over the three days preceding the day of sampling.

### Sample collection

A clinician collected three high vaginal specimens at each visit, using flocked synthetic swabs (COPAN Innovation, Italy). Two swab specimens were used for quantitative PCR for vaginal bacterial species testing [24] and the third for prostate-specific antigen (PSA) testing. The swabs were stored at 2–8°C until transport to the laboratory, where they were stored dry at −20°C until testing.

For cervicovaginal lavage samples, 10 ml normal saline at room temperature was flushed using a sterile pipette over the cervix and the lateral vaginal walls. This fluid was aspirated from the posterior fornix using the same pipette and collected in a 15 ml falcon tube that was then put in a cool box with ice (2–8°C). To collect endocervical cells, a flocked swab was inserted into the endocervical canal and gently turned over 360°. The swab was then removed and placed in a falcon tube with 10 ml phosphate buffered saline (PBS) with 1% foetal calf serum (FCS), L-glutamine (200 mM) and penicillin/streptomycin (10.000 U/ml). This procedure was repeated with a second flocked swab that was placed in the same tube as the first one. The samples were stored in a cool box together with the CVL samples and immediately transported to the laboratory for processing.

### Sample processing

Sample processing was started within one hour of sample collection for CVL and within 30 minutes for endocervical cell samples. CVL samples were centrifuged at 1000× g for 10 minutes at 4°C to get rid of debris and the supernatant (∼9 ml) was aliquoted into five fractions of approximately 1.8 ml each and stored at −80°C. Processing of the endocervical swab for cellular markers involved addition of 50 µl of 1M DL-Dithiothreitol (Sigma-Aldrich, Belgium) to the 10 ml medium and incubation for 15 minutes at 37°C to dissolve the mucus. The tube was then gently vortexed to release the cells from the swab tips, after which the swab tips were removed and cells centrifuged at 1000× g for 10 minutes at 4°C. After leucocyte and haemoglobin testing using dipsticks, the supernatant was discarded and the cell pellet resuspended in 1 ml cell culture medium. Twenty five microliters was then removed for cell counting before analysis was done by flow cytometry.

### Clinical and laboratory diagnostic tests

Estimates of the pH of vaginal secretions were determined by the study doctor using colour-fixed indicator sticks pH-Fix 3.6–6.1 (Macherey-Nagel GmbH & Co KG, Duren, Germany). These pH strips are colour-coded with graduations at pH points 3.6, 4.1, 4.4, 4.7, 5.0, 5.3, 5.6 and 6.1 and sample pH values are determined by comparing the test fields to the colour block (accuracy ±0.1 pH). Vaginal smears were examined using the Nugent scoring system in which a Nugent score of 7–10 is consistent with BV, 4–6 is an intermediate score and 0–3 reflects a normal vaginal microbiome [25]. Participants were also tested for the following sexually transmitted infections (STIs): Trichomoniasis and Candidiasis using wet mount analysis; *Chlamydia trachomatis* – CT and *Neisseria gonorrhoeae* – NG using commercial nucleic acid amplification assays.

PSA testing was performed on vaginal secretions using the Seratec® PSA semiquant assay (Seratec Diagnostica, Göttingen, Germany). A volume of 500 µl of PSA buffer was added to the thawed swab and was shaken for 2 hours. After centrifugation of 300 µl for 1 min at 13000× g, 200 µl of supernatant was used for testing according to the manufacturer's instruction.

The presence of leucocytes (sensitivity, 20–25 cells/µl as trace) and haemoglobin (sensitivity, 10 red blood cells/µl) in the supernatant of endocervical cells samples was tested using the 5+ NL Servotest® test strips (Servoprax® GmbH, Wesel, Germany).

### Cytokine and chemokine measurement

Concentrations of the inflammatory cytokines Interleukin-1α (IL-1α), IL-1β, IL-6 and IL-12(p70); anti-inflammatory cytokine IL-1 receptor antagonist (IL-1RA); CC chemokine macrophage inflammatory protein 1 beta (MIP-1β); CXC chemokines IFN-γ –induced protein (IP-10) and IL-8; growth factors granulocyte-macrophage colony-stimulating factor (GM-CSF) and granulocyte colony-stimulating factor (G-CSF) in CVL samples were analysed using the Bio-Plex™ human cytokine assay kit (Bio-Rad Laboratories NV-SA, Nazareth, Belgium) according to the manufacturer's instructions. Briefly, the lyophilized standard was reconstituted for 30 minutes on ice with 500 µl PBS containing 0.5% bovine serum albumin (BSA) and then serially diluted (1 in 4). Magnetic beads coupled with unique capture antibodies were then prepared in assay buffer and kept on ice. The assay plate was pre-wetted with 100 µl assay buffer and drained using vacuum filtration. The coupled beads were vortexed for 30 seconds and 50 µl added to each well in the assay plate. After two plate washes with 100 µl wash buffer, 50 µl of standards, samples and controls (all containing 0.5% BSA) were added to each well in the assay plate. The plate was then sealed, covered with aluminium foil and incubated on a shaker (500 rpm) at room temperature for 30 minutes. After this period, the plate was washed thrice with 100 µl wash buffer using vacuum filtration and 25 µl of biotinylated detection antibodies added to each well. The plate was sealed, covered with aluminium foil again and incubated under the same conditions as before for 30 minutes. After three washes, 50 µl of streptavidin-PE was added to the wells and incubated on a shaker (500 rpm), at room temperature for 10 minutes. After incubation, the plate was washed 3 times as before, 125 µl assay buffer added to each well, covered and shaken for 4 minutes at 500 rpm. Fluorescence data was collected using the Bio-Plex™ array reader and the Bio-Plex™ Manager 5.0 software used to calculate cytokine concentrations using a weighted five-parameter logistic curve-fitting method on the four-fold dilution series of the standard provided with the kit.

Elafin and β-defensin were measured by ELISA kits from R&D Systems (Minneapolis, MN) and Phoenix Pharmaceuticals (Burlingame, CA), respectively, following manufacturers' instructions. Optical densities were read at 450 nm with a second reference filter of 570 nm using a Victor2 multilabel reader and WorkOut Software (PerkinElmer, Waltham, MA). For the elafin assay, all CVLs were tested at a 250-fold dilution in duplicates (samples were pre-diluted in PBS with 1% BSA and then diluted 5-fold directly on the plate using the manufacturer's supplied reagent diluent). For the β-defensin assay, all CVLs were tested at a 100-fold dilution. The samples were pre-diluted 25-fold in 1% BSA/PBS and then diluted 4-fold on the ELISA plate using the manufacturer-supplied assay diluent. Samples with values below or above the assay detection ranges were repeatedly tested at lower or higher dilutions to obtain accurate protein measurements. A quality control (QC) sample was prepared by pooling CVL samples and running an aliquot of the QC pool on each elafin and β-defensin plate to assess inter-assay reproducibility. The inter-batch CV% of the QC values was 9% for the elafin ELISA kits and 18% for the β-defensin ELISA kits. The intra-assay CV% (mean +/−SD) assessed for duplicate samples measurements was 6.8+/− 5% for the elafin and 6.6+/− 7.8% for the β-defensin assay. Elafin and β-defensin concentrations were normalized to total protein determined by a BCA assay (Thermo Scientific, Rockford, IL) using the Victor 2 counter. For total protein, all samples were tested in duplicates at a 5-fold dilution in PBS and retested either undiluted or 5-fold diluted if values were below or above detection range, respectively. The total protein intra-assay CV% was 0–10% (mean +/− SD  = 2.4+/−2%).

### Flow cytometry

Endocervical cells were transferred into FACS tubes at 2×10^5^ cells per tube and spun at 590×g for 5 minutes before the supernatant was removed. Direct staining was done by incubating the cells with labelled antibodies for 20 to 30 minutes at 4°C. All antibodies were from BD-biosciences, unless otherwise stated. The following antibody-combination was used: CD14 APC (1.98 µg/ml)/Viaprobe (PerCP)/HLA-DR FITC (0.99 µg/ml)//CD3 PE (0.48 µg/ml). After washing in PBS, the cells were fixed with 1% paraformaldehyde solution.

When the number of cells was sufficient, the same samples were also used for indirect staining. Cells were first incubated with primary antibody (anti-CCR5 [20 µg/ml]; Biolegend) for 30 minutes on ice before being washed in PBS and incubated for 20 minutes with secondary antibody (biotin labelled goat anti-mouse [20 µg/ml]). After another washing step, streptavidin-PE [20 µg/ml] was added. The samples were further washed and mixed with diluted mouse serum. Finally, cells were directly labelled with antibodies before FACS analysis using a BD FACScalibur instrument. The following antibody-combinations were used: CD3 APC (1.98 µg/ml)/Viaprobe (PerCP)/CD4 FITC (0.12 µg/ml)/CCR5 PE and CD14 APC(1.98 µg/ml)/Viaprobe (PerCP)/CD4 FITC (0.12 µg/ml)/CCR5 PE. Isotype controls were used to set gates. For analysis, the cells were gated on the population that was CD3+ or CD14+. Analysis was done using the FlowJo software (version 8.8.4 TreeStar, Inc., Ashland, OR, USA).

### Bacterial species quantification

Quantitative PCR for total *Lactobacillus* species, *L. crispatus, L. iners, L. jensenii*, *L. gasseri, G. vaginalis*, and *A. vaginae* were performed as described in Jespers *et al* [24]. Briefly, the primers were synthesized by Eurogentec, Seraing, Belgium. The 25 μl PCR mixture contained QuantiTect SYBR Green PCR (Qiagen, Venlo, the Netherlands) with the exception of the PCR mixture for *L. vaginalis* which contained Thermo Scientific Absolute SYBR Green Mix (ABgene, Epsom, UK), 5 μl DNA extract, primers, and Milli-Q water. The amplification reactions were performed using the Corbett Life Science Rotor-Gene™ 6000 (Qiagen, Venlo, the Netherlands). For each of the organisms standard curves were constructed. A total of 6 standards were prepared by a tenfold dilution and within a range of 10^2^ copies/5 μl to 10^7^ copies/5 μl. The quantitative result obtained with the qPCR was expressed in number of copies/5 μl and was back calculated taking into account the total specimen elute volume, the volume extracted, the DNA extract volume obtained, and volume of DNA amplified.

### Data analysis

The lower and upper limits of quantitation for each soluble marker were defined as the lowest and highest concentration of their standards within acceptable recovery ranges (70–130%). CVL samples with soluble marker concentrations below the lower detection limit in the Bio-Plex™ assay were assigned concentrations midway between the lower limit of quantitation (LLOQ) and zero. Those above the upper detection limit were assigned values twice the upper limit of quantitation (ULOQ). In the analyses for associations with different factors that could influence the expression of these soluble markers in the FGT, log_10_ transformed values were used. To characterize the variation of the soluble markers over time between and within women, we calculated the intra-class correlation coefficient (ICC) for the analytes using the random effects model with the equation ICC  =  sigma_B_^2^/(sigma_B_^2^+sigma_W_^2^) where sigma_B_^2^ is the variance between women and sigma_W_^2^ is the within woman variance in log-transformed soluble marker concentrations. A high ICC in this context means that there is relatively more inter-woman soluble marker concentration variation than intra-woman variation i.e. the analytes are more constant for each woman compared to the total variation. ICC values were not calculated for IL-12 and GM-CSF because of the high percentage of samples that were below the detection limit.

We modelled analyte concentrations based on presence of ectopy, recent sexual activity as determined by PSA detection, menstrual cycle phase, haemoglobin presence, leucocyte presence as well as the presence of specific bacteria in the vaginal cavity and percentages of cervical cellular markers. All analyses were carried out using mixed effect linear (IL-1α, IL-1β, IL-6, IL-1RA, MIP-1β, IP-10, IL-8, G-CSF, elafin and β-defensin) or logistic regression models (for IL-12(p70) and GM-CSF) with random effects for women and fixed effects for assay plate to correct for inter-assay variability. Logistic regression models were used for IL-12(p70) and GM-CSF because 47% and 67% of the samples respectively were below the detection limit precluding means-based analyses. Each predictor was assessed for association with soluble marker concentration or presence and all significant variables were then included in a multiple predictor model. The model was simplified using stepwise exclusion until only the significant predictors remained in the model. All analyses were carried out using STATA software (version 11 College Station, Texas, USA) and graphs plotted using GraphPad Prism software (version 5.02, GraphPad Prism Software, San Diego, California, USA).

## Results

### Cohort demographics

Cohort characteristics are presented in Table 1. Four of the women (13%) had a sexual preference for the same gender and all of those self-reported to be sexually active during the study. Of the remaining 26 women with a male sexual partner preference, 69% reported sexual activity during the study. Ectopy, classified as small, moderate or large by the study doctor following a standardized protocol [26], was present in 20 out of the 30 women enrolled in the study. Seven of the 20 cases were classified as small (Table 1). After screening, 84 (60%) of the total visits were during the follicular phase (day 7) of the menstrual cycle and 57 (40%) during the luteal phase (day 21).

**Table 1:** Demographic, clinical exam and behavioural data of study population (N = 30).

Demographic, clinical exam and behavioural data	
	N (%)
**Race**	
Caucasian	30 (100)
**Sexually active during study**	
Yes	22 (73)
No	8 (27)
**Contraception**	
None	12 (40)
Intrauterine cupper device	1 (3)
Condoms	17 (57)
**Cervical ectopy**	
Absent	10 (33)
Small	7 (23)
Moderate	12 (40)
Large	1 (4)
**Partner preference**	
Male	26 (87)
Female	4 (13)
**Age** average (range)	27 (19 – 38)

### Clinical and laboratory diagnostic tests

Twelve samples (8.5%) were found to be PSA positive (Table 2), an indication of recent sexual activity. All but one of the women with positive PSA tests self-reported sexual activity during the study period. Leucocytes were detected in the supernatant of 52% and haemoglobin in 42% of the endocervical cells samples taken (Table 2). All women enrolled in the study were asymptomatic for vaginal infections with negative lab results (Trichomoniasis, Candidiasis, CT and NG) during screening and all but one of them did not have BV during the follow-up visits. Intermediate Nugent scores (4 and 6) were registered at single time points (visit 4) for two different participants (participant 33 and participant 21, respectively). Even though a Nugent score of 6 is, by definition, intermediate, participant 21 was classified as having BV based on her clinical presentation and the raised pH of 6.1.

**Table 2:** Clinical laboratory data from samples of study population (N = 141).

Clinical lab data		
		No. of samples (%)
**Vaginal secretions**	**pH**	
	3.6	98 (69.5)
	4.1	42 (29.8)
	6.1	1 (0.7)
	**Nugent score**	
	0	133 (94.3)
	1	5 (3.6)
	2	0 (0.0)
	3	1 (0.7)
	4	1 (0.7)
	5	0 (0.0)
	6	1 (0.7)
	>6	0 (0.0)
	**PSA positive**	
	Yes	12 (8.5)
	No	129 (91.5)
**Cells supernatant**	**Leucocyte presence** b	
	Yes	70 (52)
	No	65 (48)
	**Haemoglobin presence** b	
	Yes	57 (42)
	No	78 (58)

b 6 missing values.

PSA: prostate-specific antigen.

### Distribution of soluble markers concentrations in CVL

**Figure 1:**
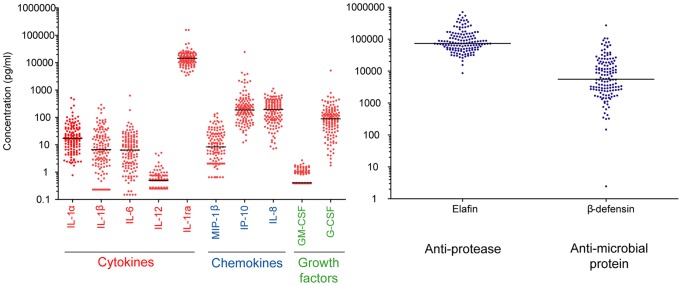
Distribution of soluble marker concentrations in lavage samples. Cytokines, chemokines and growth factors were measured in CVL samples using the Bio-Plex™ assay while Elafin and β-defensin were quantified using ELISA. Each data point represents a single sample and the line through data points represents the median concentration.

As can be seen in figure 1, the most readily measurable cytokines, chemokines and growth factors (Figure 1A) in CVL showed a median concentration in the pg/ml protein range while the anti-inflammatory IL1-RA (Figure 1A), the anti-protease elafin as well as β-defensin (Figure 1B) were in the ng/ml range. All analytes evaluated were detected in the majority of the CVL samples, except for IL-12(p70) and GM-CSF that were below the LLOQ in at least 40% of the samples. Details of the mean, median, range and SD as well as percentages detected for each soluble marker in the women in our cohort are given in table 3.

**Table 3:** Soluble markers concentrations in CVL samples of study population.

Soluble marker	Percentage detected	CVL concentration (pg/ml)
***Pro-inflammatory cytokines***		
**IL-1α**	99	17.51 (0.77 – 513.04) a 35.73 (67.27) b
**IL-1β**	90	6.73 (0.23 – 289.98) 23.05 (44.19)
**IL-6**	96	6.70 (0.15 – 624.10) 17.08 (57.33)
**IL-12**(**p70**)	53	0.50 (0.25 – 5.10) 0.61 (0.71)
***Anti-inflammatory cytokines***		
**IL-1RA**	99	14,424 (3,372 – 156,688) 16,119 (14,554)
***CC chemokines***		
**MIP-1β**	76	8.40 (0.65 – 140.90) 18.65 (25.48)
***CXC chemokines***		
**IP-10**	100	185.55 (12.23 – 24,659) 592.47 (2,247)
**IL-8**	100	203.80 (7.20 – 1,128) 247.12 (204.90)
***Growth factors***		
**GM-CSF**	33	0.405 (0.39 – 2.71) 0.68 (0.45)
**G-CSF**	100	93.36 (1.75 – 5,138) 166.74 (460.74)
***Antimicrobial proteins***		
**Elafin**	100	73,465 (8,591 – 704,092) 117,706 (116,832)
**β-defensin**	100	5,553 (2.45 – 269,839) 16,216 (29,961)
**Total protein** c	100	0.1724 (0.012 – 2.084) 0.246 (0.2312)

a Median (minimum – maximum).

b Mean (SD).

c mg/ml.

### Longitudinal variation of soluble markers concentrations in CVL

Inter- and intra-woman variation in soluble marker concentration differed from one analyte to another. For most of the soluble markers the inter-woman variation was higher than the variation between repeated samplings at different time points for the same woman (ICC >0.50). As seen in table 4, the ICC values were highest with IL-8, elafin and β-defensin meaning that for these three analytes, their concentrations were relatively more constant over time in each woman and the inter-woman variation was higher. G-CSF had the lowest ICC value (0.41) indicating a relatively large variation between repeated sampling for the same woman compared to the variation in the analyte concentrations between women. Longitudinal trends for each analyte and each woman are shown in Figure S1.

**Table 4:** Intra-class correlation coefficients (ICC) for soluble markers concentrations in CVL.

Soluble marker	ICC
Il-1α	0.60
IL-1β	0.59
IL-6	0.51
IL-12	ND a
IL-1ra	0.50
MIP-1β	0.61
IP-10	0.53
IL-8	0.70
GM-CSF	ND a
G-CSF	0.41
Elafin	0.72
β-defensin	0.63

a Not done due to the high percentage of samples in which the cytokine concentrations were below the detection limits of our assay.

### Characterization of immune cellular markers in the endocervical canal

**Figure 2:**
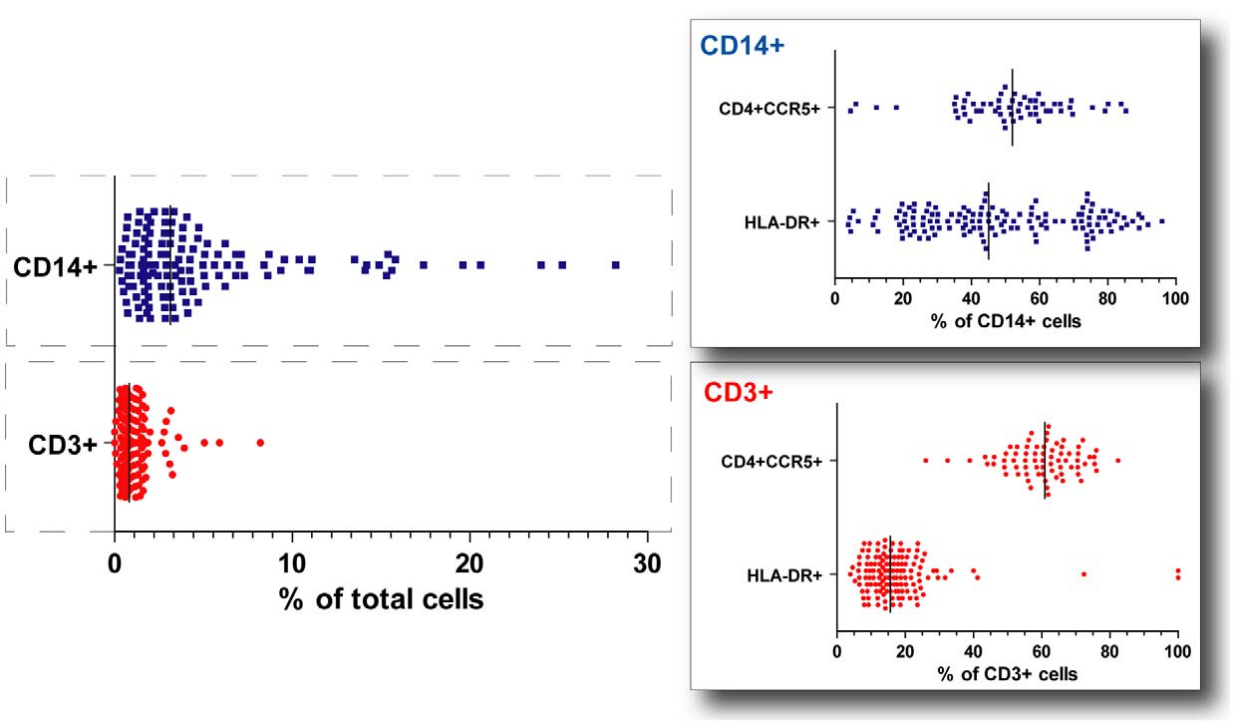
Characterization of endocervical leucocyte markers on cells sampled using flocked swabs. Three point one percent of endocervical cells collected using swabs were monocytes. Fifty two percent of these cells expressed both the CD4 and CCR5 receptors. Of the total cells, 0.8% were T-cells with 60.9% combined CD4 and CCR5 expression. Even though the women in our study were asymptomatic, a significant proportion (45.7%) of the monocytes and 15.7% of the T-cells collected from their FGT expressed the immune activation marker HLA-DR. All percentages refer to median levels in the study population.

A median of 3.1% of endocervical cells collected using swabs from the study population were monocytes of which 52% expressed both CD4 and CCR5 receptors (Figure 2). Of the total cells, a median of 0.8% were T-cells with 60.9% combined CD4 and CCR5 expression. Even though the women in our study were asymptomatic for FGT infections, a significant median proportion (45.7%) of the monocytes and 15.7% of the T-cells collected from their FGT expressed the immune activation marker HLA-DR.

### Predictors of concentrations of soluble markers in CVL

#### Clinical predictors

Cervical ectopy was strongly and consistently associated with higher levels of the pro-inflammatory IL-1β, IL-8, IL-6, MIP-1β and G-CSF soluble markers in CVL (Table 5).

**Table 5:** Association coefficients between soluble markers and their clinical, vaginal microbiome and endocervical cellular predictors.

	IL-1α	IL-1β	IL-6	IL-12^a^	IL-1RA	MIP-1β	IP-10	IL-8	GM-CSF^a^	G-CSF	IL-1RA:IL-1(α+β)	Elafin	β-defensin
**Cervical ectopy**		0.75*	0.89*			0.56*		0.48*		0.67*			
**PSA presence**			0.36^$^				0.30*						
**Menstrual cycle phase** b	0.23*				−0.08*	−0.14*	−0.13^$^				−0.24*		0.12*
**Haemoglobin**					0.07*								
**Leucocytes**	0.24*	0.40*			−0.13^$^		−0.25*			0.31*	−0.36*		
***L. crispatus*** ** & ** ***L. jensenii*** ** presence**													
***L. iners*** ** presence**		0.52^$^						0.25*					
***L. gasseri*** ** presence**									3.36*				
***G. vaginalis*** ** & ** ***A. vaginae*** ** presence**			−0.46^$^										
**% T-cells**					0.05*				1.94*			0.04*	
**% Monocytes**													
**CD3+HLADR+**													
**CD14+HLADR+**													
**CD3+CD4+CCR5+**										0.01^$^		−0.01*	
**CD14+CD4+CCR5+**													

a Associations between IL-12(p70), GM-CSF and their predictors were modelled using logistic regression and their association in the table described using odds ratios.

b Luteal (day 21) vs. follicular (day 7) phase.

Data on soluble marker concentrations was log-transformed before analysis. *Association coefficients representing associations that remained statistically significant in multivariate models. ^$^ Coefficients representing univariate associations.

PSA: prostate-specific antigen.

The levels of IL-1α and β-defensin were elevated in the CVL of women at day 21 of their menstrual cycle compared to day 7. In contrast, day 21 samples had lower IL-1RA and MIP-1β concentrations compared to day 7 samples. Lower levels of IP-10 were also observed in day 21 samples with statistical significance only in the univariate regression model.

In CVL samples of women with detectable white blood cells, higher levels of IL-1α, IL-1β and G-CSF and lower levels of IP-10 were quantified.

The presence of PSA in vaginal secretions and the presence of haemoglobin in endocervical cells' supernatant showed single, independent associations with higher IP-10 and IL-1RA concentrations in CVL, respectively.

#### Vaginal microbiome predictors

No significant associations were observed between any of the species over more than one soluble marker (Table 5). Single associations were observed between the presence of *Lactobacillus iners*, a non-H_2_O_2_ producer and IL-8. GM-CSF appeared to be higher in samples from women with *Lactobacillus gasseri.* Women with *Lactobacillus crispatus* and *Lactobacillus jensenii* present showed a negative association with cellular inflammatory markers (Table 6) but no association with soluble inflammatory markers.

**Table 6:** Association coefficients between endocervical cellular markers and their clinical and vaginal microbiome predictors.

	% T-cells	% Monocytes	CD3+ HLADR+	CD14+ HLADR+	CD3+ CD4+ CCR5+
**Cervical ectopy**		2.67*		−16.81*	8.49*
**PSA presence**					
**Menstrual cycle phase** a		1.95*	5.27*	−7.97^$^	5.02*
**Haemoglobin**				9.38*	
**Leucocytes**					7.5^$^
***L. crispatus*** ** & ** ***L. jensenii*** ** presence**	−0.448*		−6.94*		−6.113*
***L. iners*** ** presence**					
***L. gasseri*** ** presence**					
***G. vaginalis*** ** & ** ***A. vaginae*** ** presence**					

a Luteal (day 21) vs. follicular (day 7) phase.

Data on soluble marker concentrations was log-transformed before analysis. No associations were found with CD14+ CD4+ CCR5+ cells. *Association coefficients representing associations that remained statistically significant in multivariate models. ^$^ Coefficients representing univariate associations.

PSA: prostate-specific antigen.

#### Endocervical cellular predictors

In the samples with higher percentages of CD3 positive T-cells, concentrations of IL-1RA, GM-CSF and elafin were also higher. Higher percentages of CD3+ CD4+ CCR5+ cells were weakly associated with lower levels of elafin.

### Predictors of expression of cellular markers in the endocervix

The presence of ectopy was associated with a higher percentage of total monocytes and CD3+ CD4+ CCR5+ cells but a lower percentage of activated monocytes (Table 6). As with soluble markers, fluctuations with the menstrual cycle was observed with the cellular markers. Specifically, total monocytes, CD3+ HLA-DR+ and CD3+ CD4+ CCR5+ cells were higher on day 21 compared to day 7 of the menstrual cycle. The presence of haemoglobin in the cells' supernatant was associated with a higher percentage of activated monocytes.

The impact of bacterial species on the cellular markers in the endocervical canal in our cohort was only seen with the presence of both *L. crispatus* and *L. jensenii* that was associated with reduced total T-cells, CD3+ HLA-DR+ and CD3+ CD4+ CCR5+ cells in our cohort.

### Participants with intermediate Nugent scores

**Figure 3:**
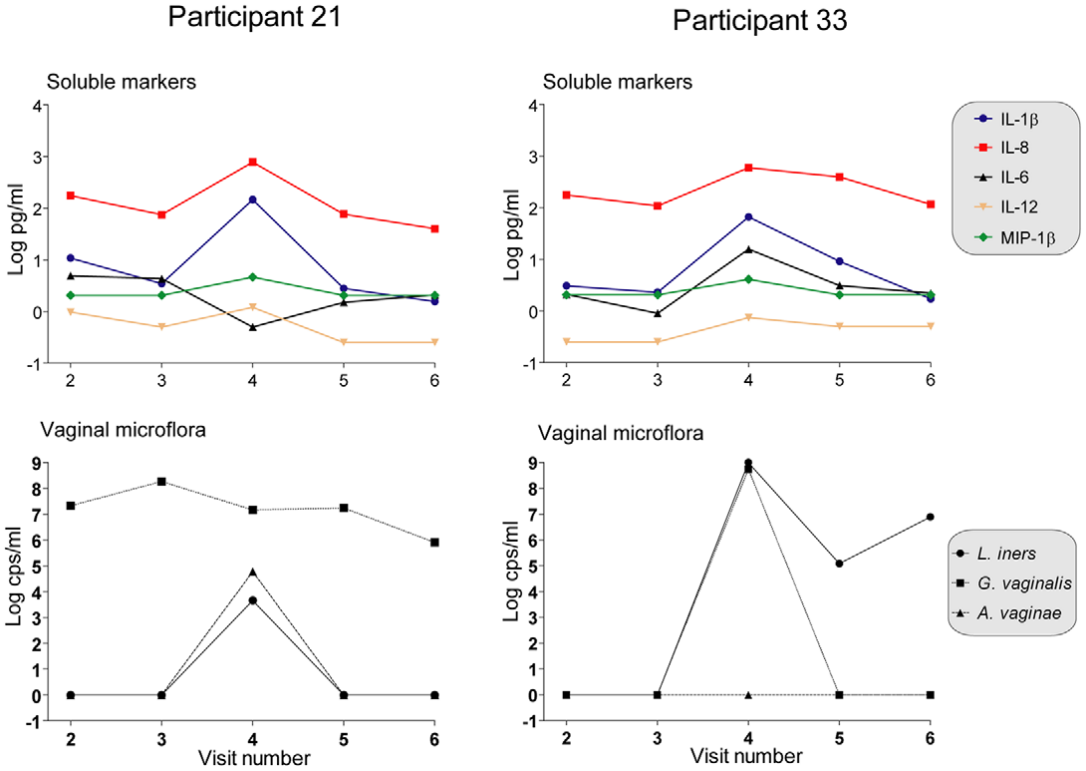
Case profiles of longitudinal trends of selected bacterial species counts and soluble biomarkers for women with intermediate Nugent scores on visit 4. Two women with intermediate Nugent scores on their fourth visits to the clinic (participants 21 and 33) had unique expression profiles of specific soluble markers and bacterial species colonization. Specifically, IL-1β, IL-8 and modestly IL-12(p70) and MIP-1β expression in these women peaked at visit 4. Interestingly, visit 4 was also the time point at which *A. vaginae* and *L. iners* peaked for participant 21 and *G. vaginalis* and *L. iners* peaked for participant 33. Of note, IL-6 expression dipped in participant 21 but peaked in participant 33 at visit four.

The two women with intermediate Nugent scores on their fourth visits to the clinic (participants 21 and 33) had unique expression profiles of specific soluble markers and bacterial species colonization. Specifically, IL-1β, IL-8, IL-12(p70) and MIP-1β expression in these women peaked at visit 4. Visit 4 was also the time point at which the highest quantity of *A. vaginae* and *L. iners* but not *G. vaginalis* were detected in the vaginal swab samples of participant 21 (Figure 3). The bacterial species profile of participant 33 was slightly different with *G. vaginalis* and *L. iners* but not *A. vaginae* peaking at visit four. Interestingly, IL-6 expression dipped in participant 21 but peaked in participant 33 at that time point.

## Discussion

Characterization of soluble and cellular biomarkers and the factors that influence their secretion in the FGT is undoubtedly important for the field of anti-HIV microbicide development. Our study describes normative expression levels of a selected panel of soluble biomarkers in the lower FGT of a typical healthy Caucasian population for an early phase I microbicide trial. These biomarkers were selected based on comparison of an initial Bio-Plex™ run with 27 analytes with results from two different populations in Rwanda and the United States of America as described elsewhere [27]. In addition we characterized cervical T-cells and monocytes, which are markers of cellular immunity, sensitive to inflammation and potentially susceptible to HIV. Finally, we investigated whether characteristics such as ectopy, menstrual cycle phase, bacterial species and the presence of PSA, leucocytes and haemoglobin in endocervical secretions were associated with levels of soluble and cellular biomarkers in the female genital mucosa.

Not all selected soluble biomarkers were detected in all samples. This observation is in agreement with other studies where, for example, GM-CSF and IL-12(p70) were also below the LLOQ for the majority of samples regardless of sampling site (endocervix or vagina) or specimen collection method [28]. Separately, in the ECS samples of female adolescents in the US collected by Weck-cel® sponges, 39% of samples analysed for IL-12(p70) were below the detection limit [29]. In a different study by Lieberman *et al* [12], however, IL-12(p70) was readily detectable in all endocervical secretion samples of healthy, non-pregnant women. These discrepant results may be reflective of methodological differences between the studies and highlight the need of developing standardised methods that should be used for analyte detection if results are to be favourably compared between studies for eventual selection of biomarkers to be used in clinical trials.

Cervical ectopy is a condition in which a proportion of the ectocervix is lined by columnar epithelium instead of the multi-layered squamous epithelium usually found in the mature ectocervix. A thinner mucosal barrier would imply greater vulnerability to physical trauma during coitus leading to inflammation and also exposure of sub-mucosal HIV target cells. It is not surprising therefore that previous studies have found ectopy to be a probable risk factor for HIV infection [30], [31], [32]. The strong association of cervical ectopy with the mainly pro-inflammatory soluble markers in our study is consistent with a recent study that found higher levels of pro-inflammatory cytokines/chemokines in the CVL of healthy young women with predominantly columnar and metaplastic ectocervical epithelium compared to predominant squamous epithelium [33] including IL-1β, IL-6 and IL-8 as observed in our study. Previously, *in vitro* experiments with non-stimulated immortalized cell lines of endocervical origin showed higher expression of IL-6, IL-8 and M-CSF compared with cell lines of ectocervical origin under the same conditions [34] corroborating observations seen in women with ectopy.

Based on a general consistency between anamnestic data and testing for PSA, this marker was found to be a reliable measure of recent sexual activity in our study population. However, the single incidence where the FGT secretions tested positive for PSA even though the subject did not report sexual activity indicates the importance of verification of self-reported data by laboratory methods especially in the context of microbicide trials. Previous reports of ectopic prostatic tissue in the upper and lower FGT [35], [36], [37] that could explain exceptional detection of PSA in semen-free vaginal samples [38] call for caution in interpretation of results. That said, intercourse is known to disrupt the vaginal ecosystem as constituents of seminal plasma optimize conditions to promote conception. For example, vaginal pH is increased after coitus due to the higher pH of semen, with semen also promoting the influx of leucocytes and Langerhans cells into the FGT [39], [40]. Changes in vaginal bacterial species can also be expected as during coitus, bacteria colonising the perineum could be transferred into the vagina. The observation in our study of increased concentrations of IP-10 (and univariately IL-6) in the presence of PSA are in agreement with a recent study that reported increased leukocyte recruitment and pro-inflammatory cytokine mRNA expression in ectocervical tissue exposed to seminal fluid during coitus [41]. This pro-inflammatory effect was independent of the physical effects of coitus as it was not seen in controls who used condoms. *In vitro*, seminal plasma induced IL-6 production in ectocervical cells but not vaginal or endocervical cells [39]. These changes, coupled with the physical effects of coitus, can lead to greater susceptibility to infection upon exposure to HIV and should also be taken into account in clinical trials that also assess the effects of microbicide compounds on the FGT.

Our study also recorded variations in concentrations of specific soluble and cellular immune modulators with the menstrual cycle. These variations are not surprising given the hormonal changes occurring in the female genital tract during the course of the menstrual cycle. Wira and Fahey even suggested a window of viral infectivity on days 14–23 of the menstrual cycle during which FGT immunity is suppressed by sex hormones [42]. In the CVL of healthy pre-menopausal women, Al-Harthi and colleagues [43] found five-fold higher expression of IL-6 and IL-1β in the follicular compared to the luteal phase of the menstrual cycle. In contrast, the same group found equal expression of the same cytokines in the CVL of HIV-seropositive pre-menopausal women with significant elevation only seen during menses [44]. Our study group did not show differences in the expression of these two cytokines between the follicular and luteal phases. While HIV infection could explain differences observed in their studies and ours, a significant difference is that our study controlled for ectopy which was associated with both IL-1β and IL-6 expression and could have confounded their analyses. Fleming *et al* [45] also demonstrated maximal β-defensin-1 mRNA expression in the endometrium during the mid-secretory phase and maximal β-defensin-2 mRNA expression during menstruation. Another study showed positive correlation of CVL levels of M-CSF with serum levels of E2 and the E2/P ratio [46] and increased levels of IL-1β and TGFβ2 [46]. Estradiol, a hormone which is increased together with progesterone in the luteal phase, has previously been shown to increase mRNA expression of human β-defensin-2 by uterine epithelial cells while at the same time inhibiting the expression of the pro-inflammatory TNF-α, IL-6 and IL-8 *in vitro* [47]. This probable two-sided effect of the sex hormone is also seen in our study where IP-10 and MIP-1β were decreased on day 21 compared to day 7 of the menstrual cycle. It remains unknown, however, what role progesterone played in these fluctuations and future *in vitro* studies would benefit from assessing the combined effects of these sex hormones to gain a better understanding of the *in vivo* situation. Of note, we demonstrated increased expression of IL-1α and β-defensin and decreased expression of IL-1RA in the luteal compared to the follicular phase of the menstrual cycle, highlighting the need to take into account the menstrual cycle time point during microbicide trials. Differential counts of *L. crispatus* (0.22 log higher) and *L. iners* (0.83 log lower) on day 21 compared to day 7 of the menstrual cycle were also observed in a sub-group of our study population as described elsewhere by Jespers *et al* [24].

Detection of haemoglobin in cervicovaginal secretions is not unusual even when sampling is done outside menses. Haemoglobin was detected in 64% of ECS by Lieberman *et al* [12] although there was no significant association with any of the analytes tested in their study. An earlier study documented significantly higher concentrations of IL-10 and IL-12(p70) in blood-contaminated ECS samples compared to non-contaminated samples [29]. Haemoglobin presence in cells supernatant was positively but weakly associated with IL-1RA and activated monocytes in our population. These results need to be interpreted with caution as it is possible that soluble and cellular markers of systemic origin leak into the FGT due to trauma from sampling and are not reflective of a local immune response at the female genital mucosa. Leucocyte presence that is a normal marker of inflammation was unsurprisingly positively associated with pro-inflammatory analytes IL-1α, IL-1β and G-CSF in CVL and negatively with IL-1RA. A recent study also found a strong correlation between the neutrophil marker myeloperoxidase (MPO) and G-CSF that supports neutrophil function [48].

Bacterial vaginosis, a condition in which the vagina is colonized by anaerobic bacteria instead of the protective lactobacilli species, has been associated with increased susceptibility to STIs and HIV infection [49], [50], [51]. In our study population, the presence of both *G. vaginalis* and *A. vaginae* was inversely correlated with IL-6. This partial dampening of the immune response could possibly explain the presence of these BV related organisms in healthy asymptomatic women. The two participants (21 and 33) with intermediate Nugent scores had distinct vaginal microbiome and soluble marker expression profiles. Participant 21 complained of a vaginal itch, had a white watery discharge on examination and a vaginal pH of 6.1 during her fourth visit to the clinic. She was treated with 150mg Diflucan for clinical candidiasis and improved on the next day. These clinical symptoms combined with a Nugent score of six led to her classification as having BV. The Nugent scores during her other visits were all zero. Interestingly, a longitudinal assessment of the CVL concentrations of the soluble immune modulators in participants 21 and 33 show similar trends (figure 3); IL-1β, IL-8 and modestly IL-12(p70) and MIP-1β all peak during visit 4 when these participants had intermediate Nugent scores. These observations are partly in agreement with a different study where both IL-1β and IL-1RA were found to be significantly higher in women with intermediate Nugent scores compared to women with normal Nugent scores [19]. Cauci *et al* [52] showed 13 fold vaginal IL-1β in women with BV in association with anti-*Gardnerella vaginalis* hemolysin (Gvh) IgA response. They concluded that the induction of the pro-inflammatory cytokine IL-1β might be a necessary event to elicit an innate immune response to control anaerobic genital tract infections and that high levels of vaginal IL-1β were associated with mounting of an antigen-specific mucosal immune response in women with bacterial vaginosis [53]. A recent *in vitro* model of vaginal bacterial colonization showed that in contrast to *L. crispatus*, BV-associated *P. bivia* and especially *A. vaginae* induce increased production of pro-inflammatory chemokines (e.g. IL-8) [54]. Importantly, the peaks in pro-inflammatory cytokines observed in these two participants in our study coincide with peaks of both *A. vaginae* and *L. iners* for participant 21 and *G. vaginalis* and *L. iners* for participant 33 (figure 3). In participant 21, IL-6 concentrations dipped during visit 4 when both *A. vaginae* and *G. vaginalis* were present corroborating the inverse association seen before. *G. vaginalis* was constantly present in this participant and she only developed clinical symptoms when *A. vaginae* was also present. In contrast, in participant 33 IL-6 peaked during this visit but there was also no peak of *A. vaginae*, suggesting that *A. vaginae* and *G. vaginalis* work in concert to dampen IL-6 expression and cause BV.

The presence of both *L. crispatus* and *L. jensenii* was not related to any of the cytokines or chemokines and had an inverse association with the total percentage of T-cells, CD3+ HLA-DR+ and CD3+ CD4+ CCR5+ cells. This is in agreement with the knowledge that the *Lactobacillus* species are the major constituents of the healthy vaginal microbiota in women and that they have consistently been associated with absence of vaginal symptoms, reduced risk of STIs and a healthy pregnancy outcome [55], [56]. In our healthy women the presence of *L. iners*, a non-H_2_O_2_ producing bacterium, was associated with increased IL-1β and IL-8. *L. iners* is present in high numbers in women with and without BV as demonstrated by Jespers *et al* [24]. In contrast to studies carried out in North America and Europe where *L. crispatus* dominates, *L. iners* has been shown to be the predominant species in a study in Nigeria [57]. Unpublished data from Tanzania also shows predominance in *L. iners* [58] in spite of a normal flora as defined by Nugent. Assuming that *L. crispatus* is the key flora of a healthy vagina, the lack of it may be related to the high BV population prevalence in Tanzania. In addition, Srinivasan and colleagues showed that concentrations of *L. iners* increase after antibiotic treatment for BV, suggesting that it fills in for bacteria successfully eradicated by treatment [59] and that women who have been previously treated for BV, may be at higher risk for recurrence. Associations between the different types of vaginal flora in this study population are described elsewhere by Jespers *et al* [24].

A potential weakness of the study is that ectopy was determined by observation and not by photography and computer-assisted measurement. The associations with ectopy however remained strongly significant in the multivariate analyses ruling out the probability that they were chance associations. Additionally, scoring of ectopy for all participants was done by a single physician using a documented manual, thereby excluding variation due to subjective interpretation/classification. Finally, statistical significance was retained in the associations even when only the moderate and large ectopies were considered as present and the small ones classified as absent (data not shown). Given the relatively large number of possible predictors and outcome measures, only associations which were observed across several soluble markers in CVL are expected to be reproducible. Single associations could be due to chance and should be confirmed in further studies.

Soluble marker concentrations described in this study can act as reference values for further studies in women with similar or different profiles. Comparison of concentrations across studies will however only be feasible once sample collection, processing and measurement methods are standardized. Most studies use traditional ELISAs or multiplex immunoassays for soluble markers detection. The main advantage of using multiplex assays for detection of soluble biomarkers is that very small volumes of samples are needed to detect multiple analytes and this is especially beneficial in the case of ECS where eluted sample volumes are limited. Establishing the normative ranges of soluble and cellular biomarkers in healthy populations allows for the selection of specific ones with distinct expression profiles under conditions of immune activation and whose variations can be universally attributed to specific predictors and hence can be reliably used as indicators of product safety within the setting of microbicides clinical trials. In future, clinical trials should consider ectopy, sexual activity as determined by PSA presence, menstrual cycle phase and presence of bacterial species in the FGT as possible confounders when evaluating the possible inflammatory effects of microbicide compounds in the FGT.

## Supporting Information

Figure S1 **Longitudinal trends of soluble markers concentrations in CVL by participant.** Longitudinal trends for each of the analytes (IL-1α, IL-1β, IL-6, IL-12, IL-1RA, MIP-1β, IP-10, IL-8, GM-CSF, G-CSF, elafin and β-defensin) for each participant are shown.
(PDF)
