# Supplementary material for: Searching for Lower Female Genital Tract Soluble and Cellular Biomarkers: Defining Levels and Predictors in a Cohort of Healthy Caucasian Women
Source: PLoS One. 2012 Aug 31;7(8):e43951. doi: 10.1371/journal.pone.0043951 (PMC3432048; doi:10.1371/journal.pone.0043951)
Supplement: Figure S1 — Longitudinal trends of soluble markers concentrations in CVL by participant. Longitudinal trends for each of the analytes (IL-1α, IL-1β, IL-6, IL-12, IL-1RA, MIP-1β, IP-10, IL-8, GM-CSF, G-CSF, elafin and β-defensin) for each participant are shown. (PDF) [file pone.0043951.s001.pdf]

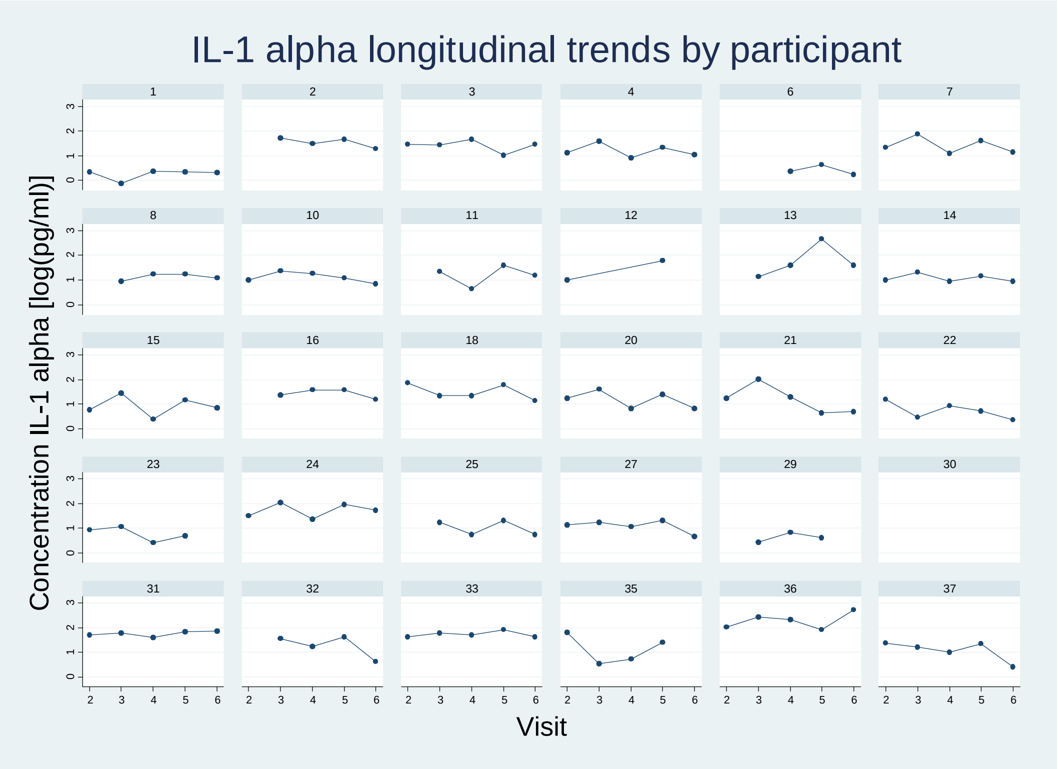
IL-1 alpha longitudinal trends by participant
Concentration IL-1 alpha [log(pg/ml)]
Visit
1
2
3
4
6
7
8
10
11
12
13
14
15
16
18
20
21
22
23
24
25
27
29
30
31
32
33
35
36
37

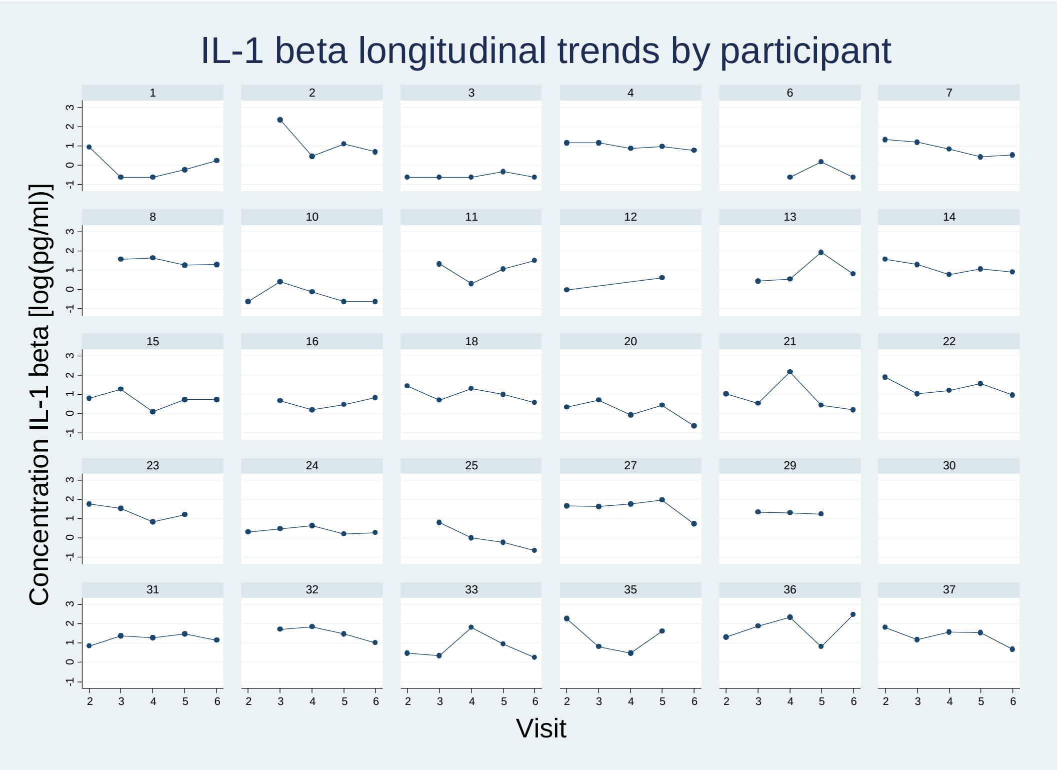
IL-1 beta longitudinal trends by participant
Concentration IL-1 beta [log(pg/ml)]
Visit
1
2
3
4
6
7
8
10
11
12
13
14
15
16
18
20
21
22
23
24
25
27
29
30
31
32
33
35
36
37

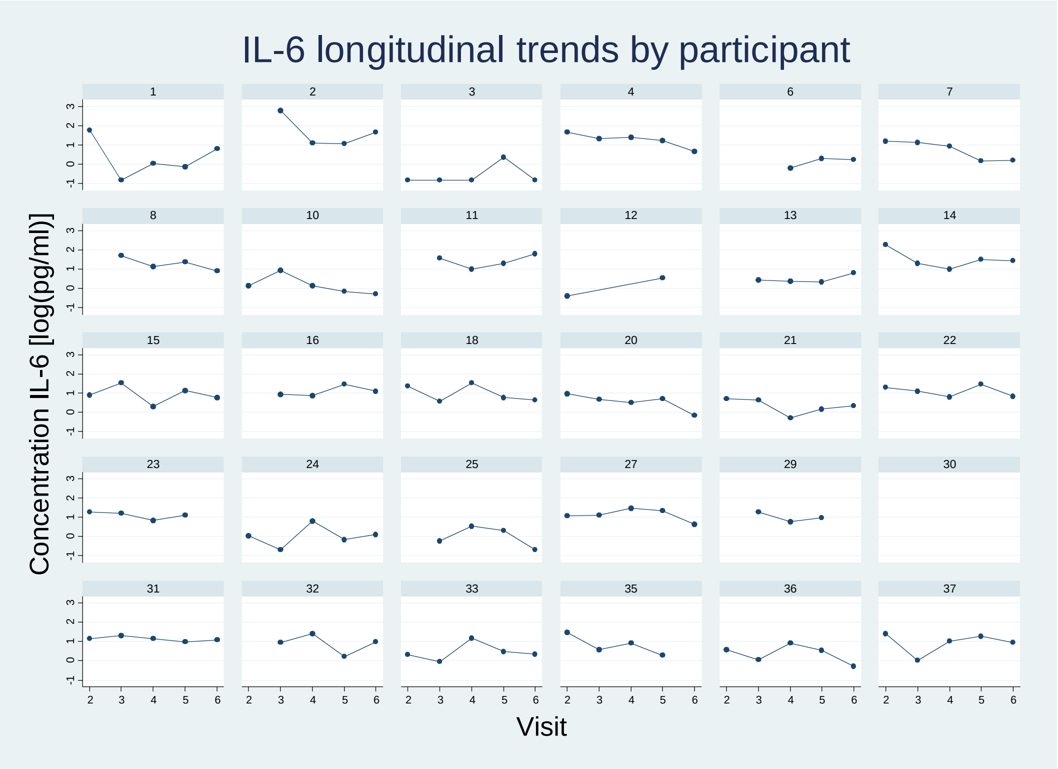
IL-6 longitudinal trends by participant
1
2
3
4
6
7
8
10
11
12
13
14
15
16
18
20
21
22
23
24
25
27
29
30
31
32
33
35
36
37
Concentration IL-6 [log(pg/ml)]
Visit

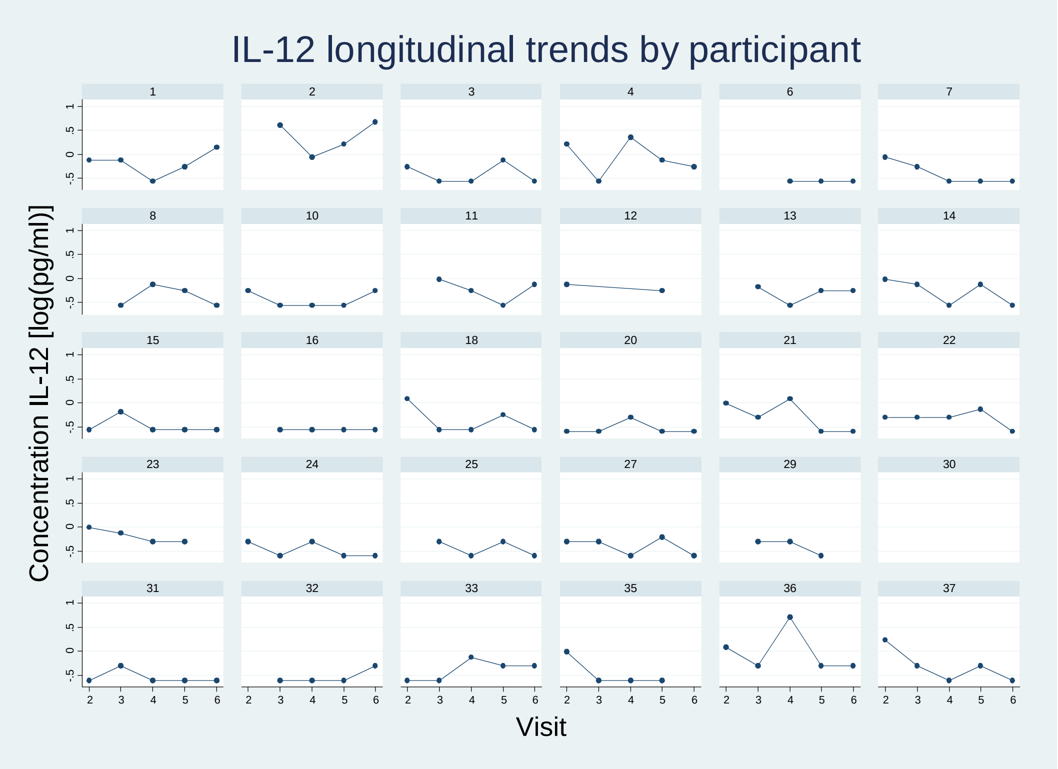
IL-12 longitudinal trends by participant
1
2
3
4
6
7
8
10
11
12
13
14
15
16
18
20
21
22
23
24
25
27
29
30
31
32
33
35
36
37
Concentration IL-12 [log(pg/ml)]
Visit

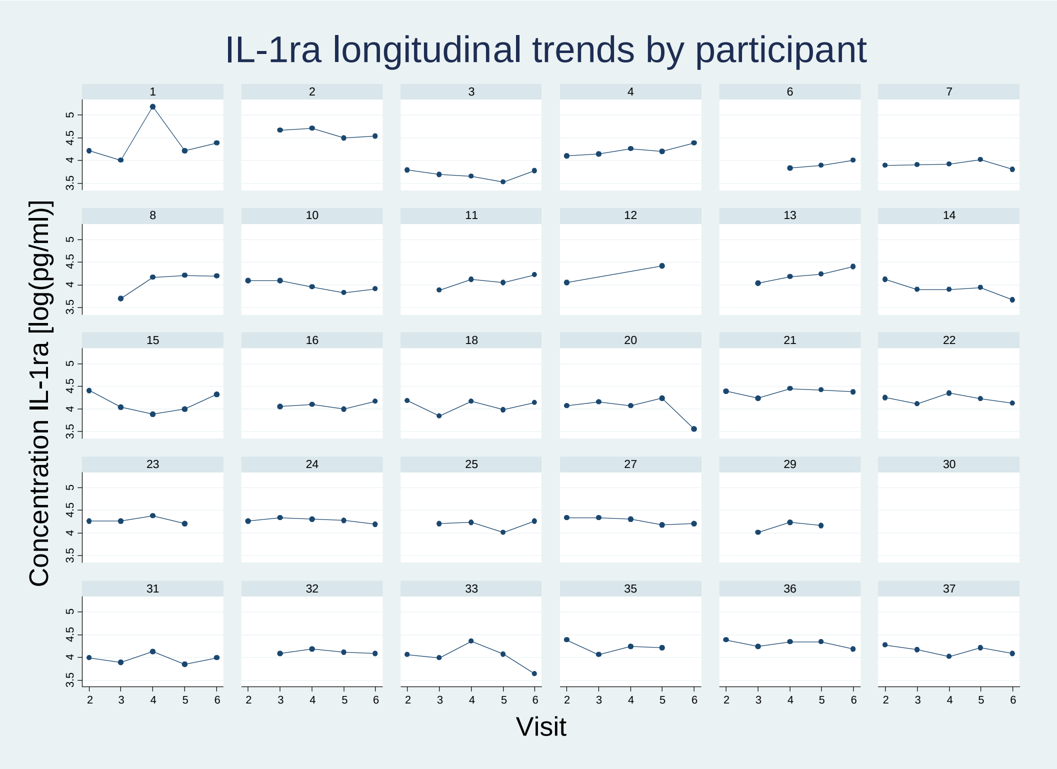
IL-1ra longitudinal trends by participant
Concentration IL-1ra [log(pg/ml)]
Visit

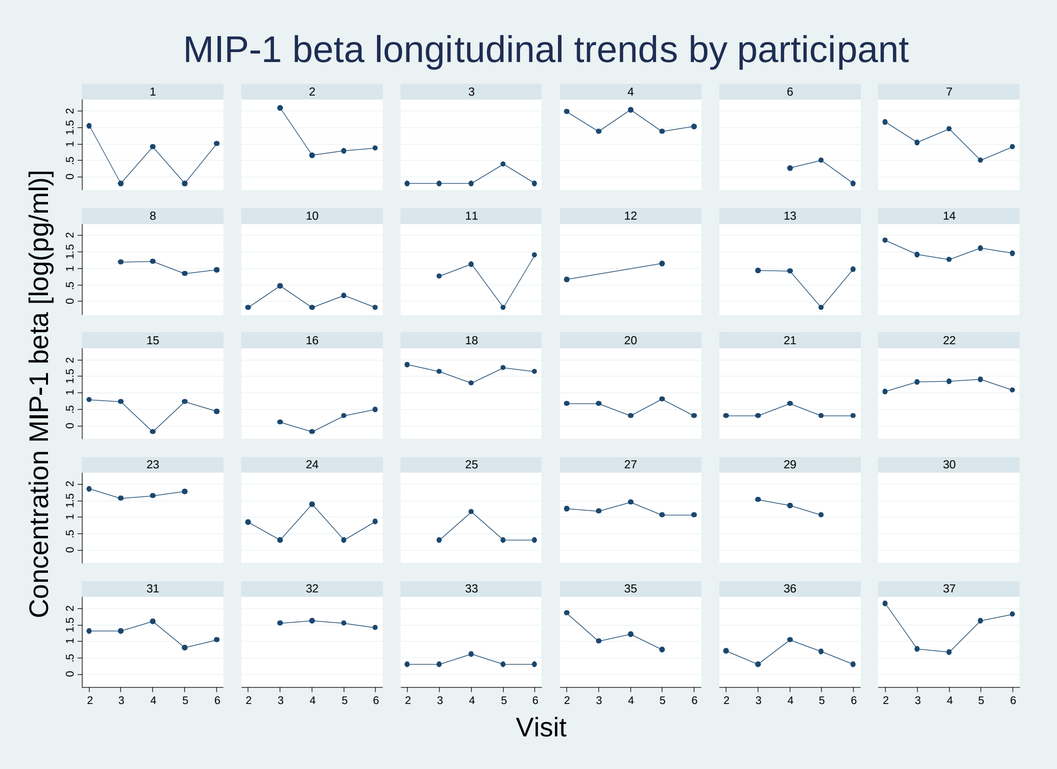
MIP-1 beta longitudinal trends by participant
Concentration MIP-1 beta [log(pg/ml)]
Visit

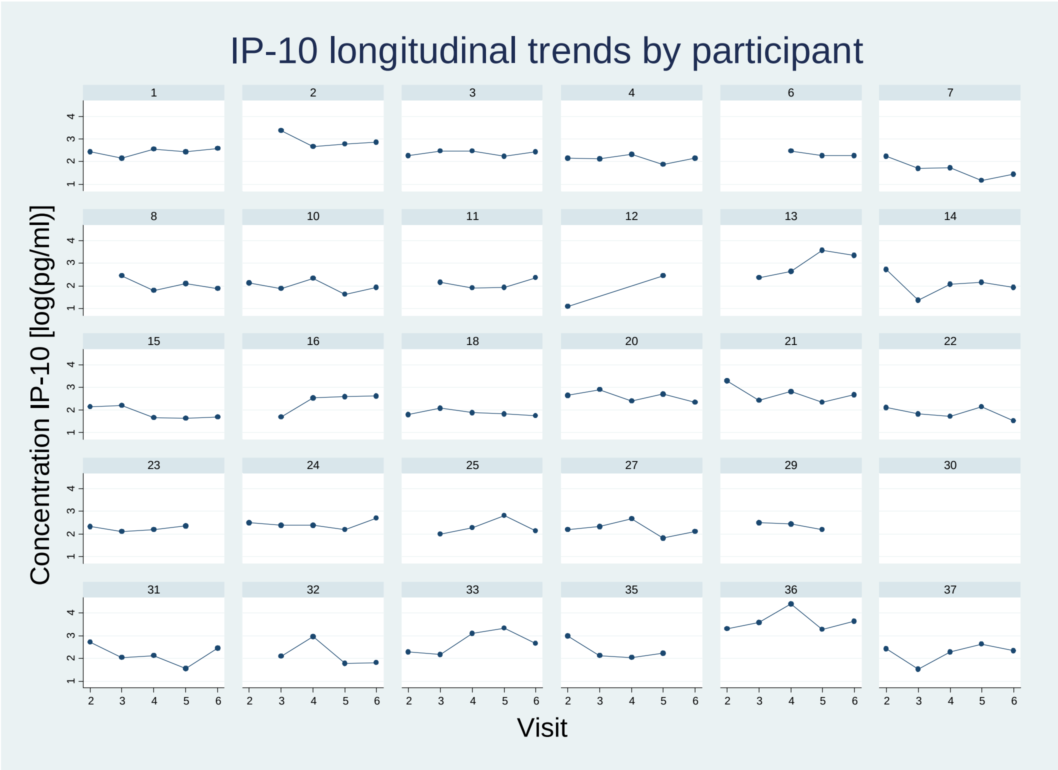
IP-10 longitudinal trends by participant
Concentration IP-10 [log(pg/ml)]
Visit
1
2
3
4
6
7
8
10
11
12
13
14
15
16
18
20
21
22
23
24
25
27
29
30
31
32
33
35
36
37

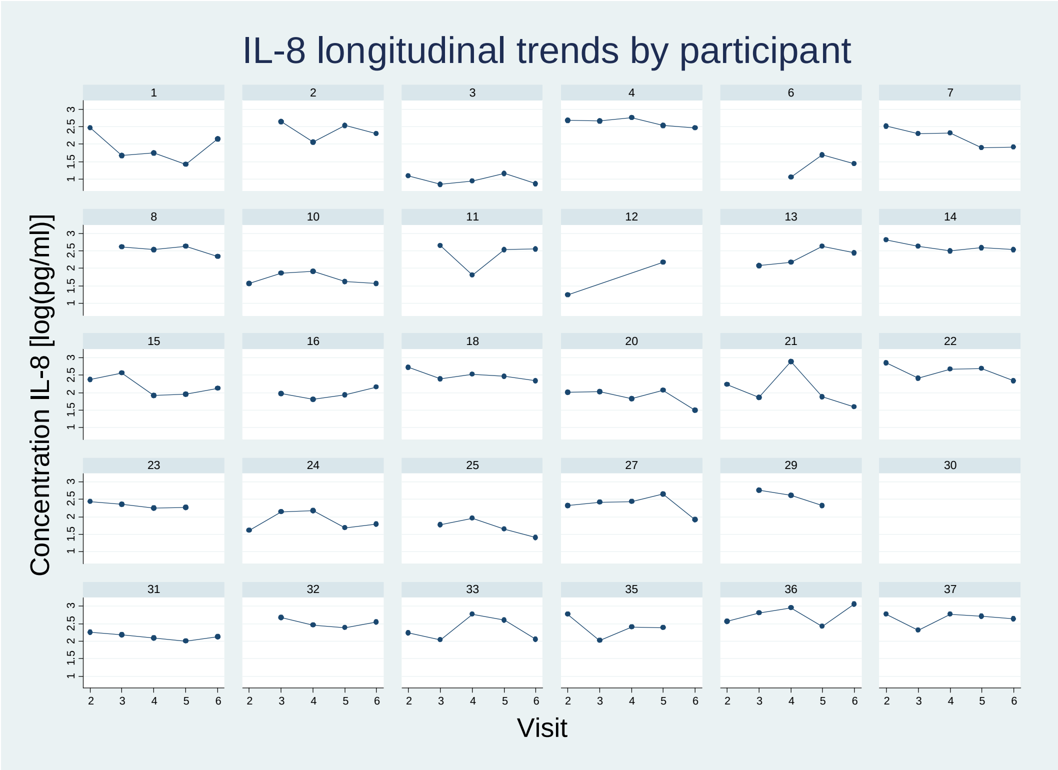
IL-8 longitudinal trends by participant
Concentration IL-8 [log(pg/ml)]
Visit
1
2
3
4
6
7
8
10
11
12
13
14
15
16
18
20
21
22
23
24
25
27
29
30
31
32
33
35
36
37

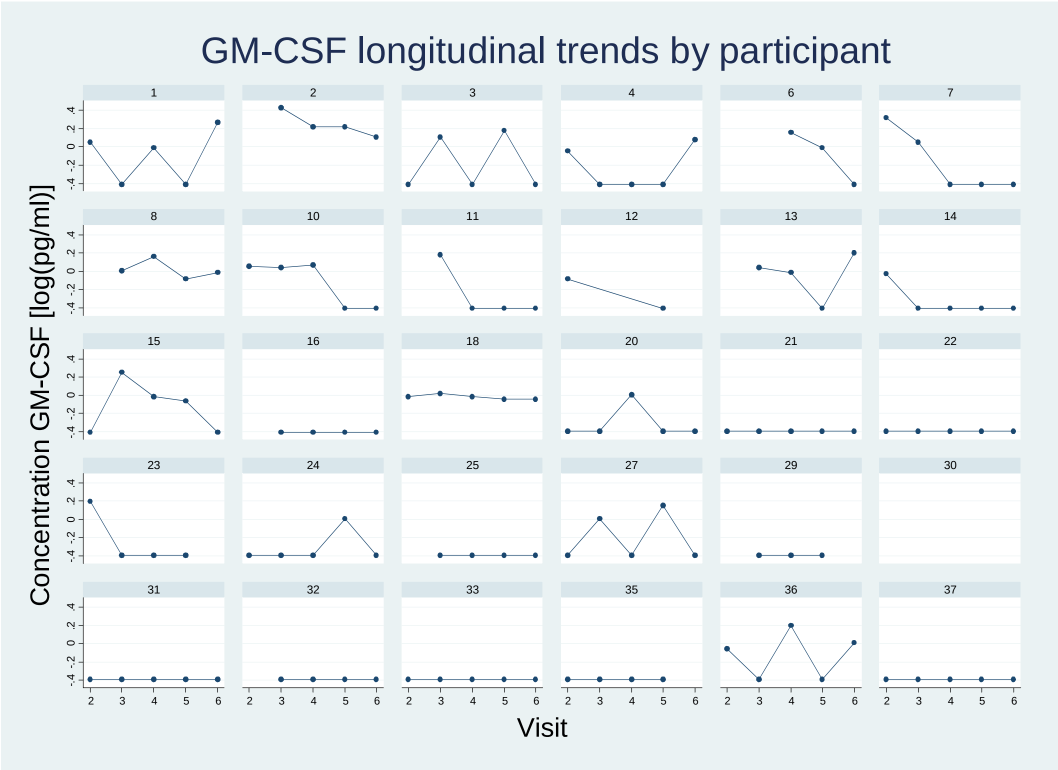
GM-CSF longitudinal trends by participant
Concentration GM-CSF [log(pg/ml)]
Visit
1
2
3
4
6
7
8
10
11
12
13
14
15
16
18
20
21
22
23
24
25
27
29
30
31
32
33
35
36
37

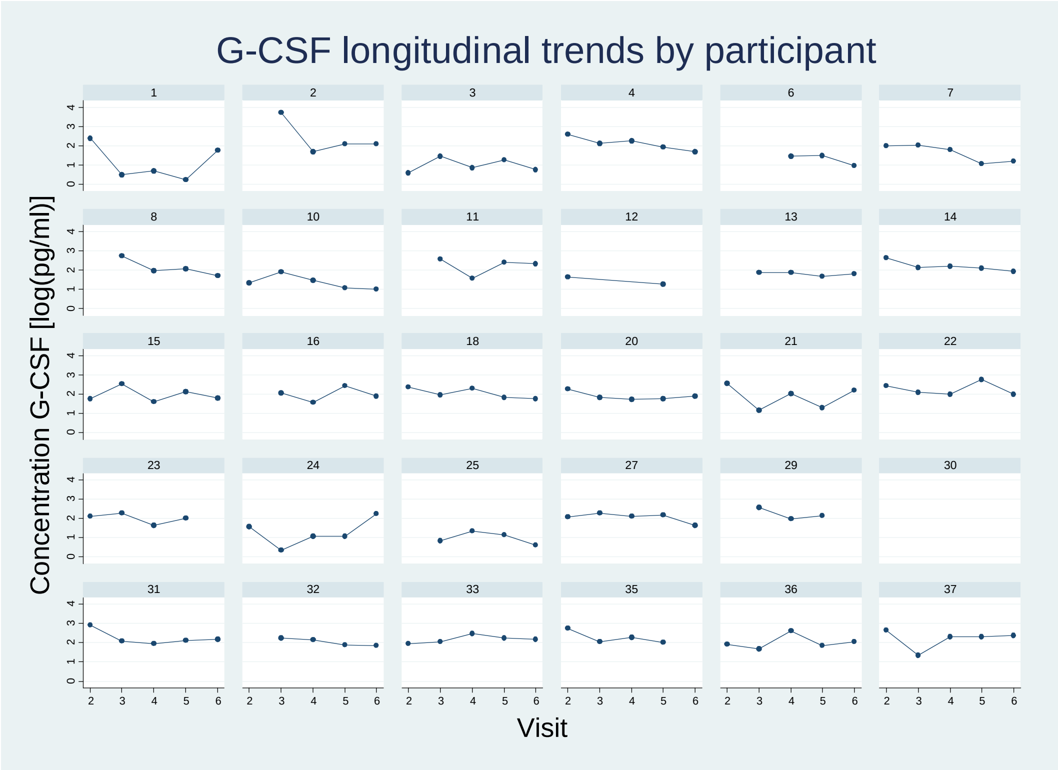
G-CSF longitudinal trends by participant
Concentration G-CSF [log(pg/ml)]
Visit
1
2
3
4
6
7
8
10
11
12
13
14
15
16
18
20
21
22
23
24
25
27
29
30
31
32
33
35
36
37

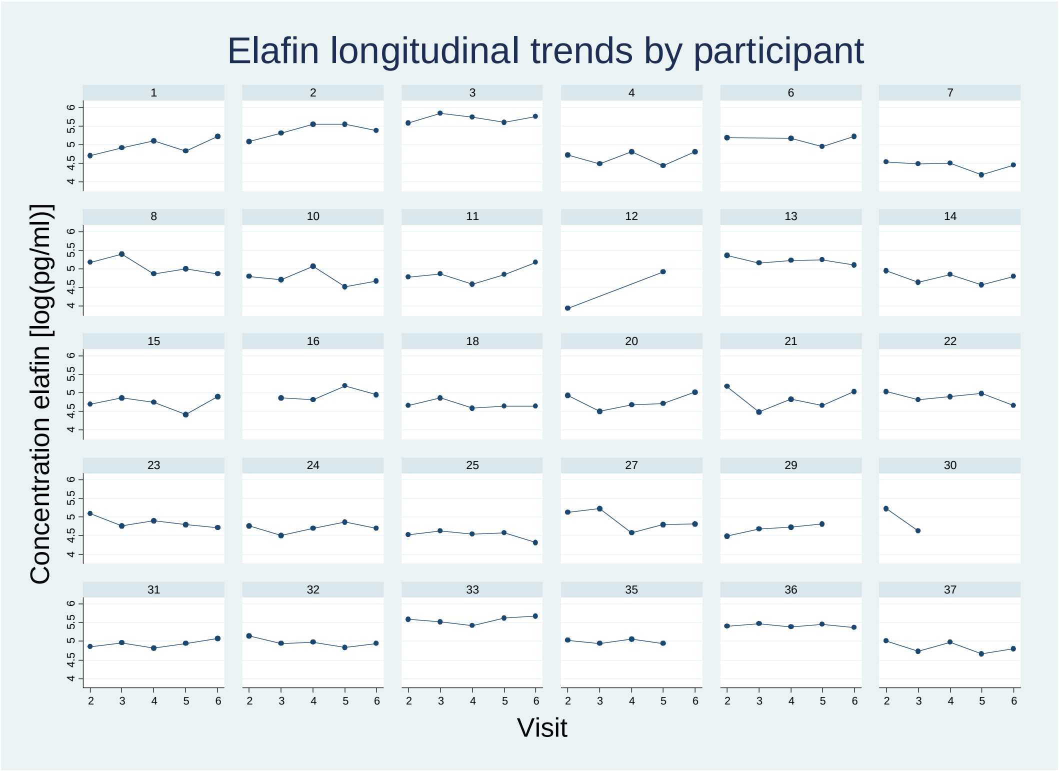
Elafin longitudinal trends by participant
Concentration elafin [log(pg/ml)]
Visit
1
2
3
4
6
7
8
10
11
12
13
14
15
16
18
20
21
22
23
24
25
27
29
30
31
32
33
35
36
37

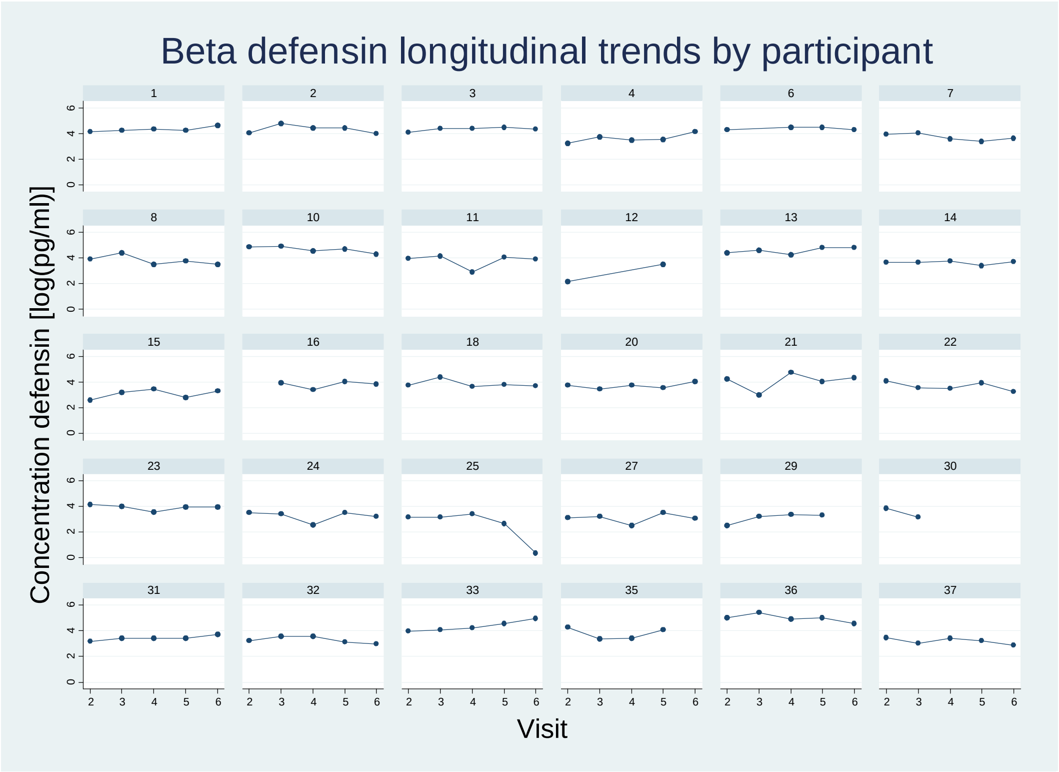
Beta defensin longitudinal trends by participant
Concentration defensin [log(pg/ml)]
Visit
1
2
3
4
6
7
8
10
11
12
13
14
15
16
18
20
21
22
23
24
25
27
29
30
31
32
33
35
36
37
